# Male contraception: narrative review of ongoing research

**DOI:** 10.1186/s12610-023-00204-z

**Published:** 2023-11-09

**Authors:** Eli J. Louwagie, Garrett F.L. Quinn, Kristi L. Pond, Keith A. Hansen

**Affiliations:** 1 University of South Dakota Sanford School of Medicine, 1400 W 22nd St, Sioux Falls, SD 57105 USA; 2 Chair and Professor, Dept. of Obstetrics and Gynecology, University of South Dakota Sanford School of Medicine; Reproductive Endocrinologist, Sanford Fertility and Reproductive Medicine, 1500 W 22nd St Suite 102, Sioux Falls, SD 57105 USA

**Keywords:** Male contraception, Reproductive health, Endocrinology, Androgens, Progestin, Spermatogenesis, Sperm motility, Vas deferens, Contraception masculine, Santé reproductive, Endocrinologie, Androgènes, Progestatif, Spermatogenèse, Mobilité des Spermatozoïdes, Canal déférent

## Abstract

**Background:**

Since the release of the combined oral contraceptive pill in 1960, women have shouldered the burden of contraception and family planning. Over 60 years later, this is still the case as the only practical, effective contraceptive options available to men are condoms and vasectomy. However, there are now a variety of promising hormonal and non-hormonal male contraceptive options being studied. The purpose of this narrative review is to provide clinicians and laypeople with focused, up-to-date descriptions of novel strategies and targets for male contraception. We include a cautiously optimistic discussion of benefits and potential drawbacks, highlighting several methods in preclinical and clinical stages of development.

**Results:**

As of June 2023, two hormonal male contraceptive methods are undergoing phase II clinical trials for safety and efficacy. A large-scale, international phase IIb trial investigating efficacy of transdermal segesterone acetate (Nestorone) plus testosterone gel has enrolled over 460 couples with completion estimated for late 2024. A second hormonal method, dimethandrolone undecanoate, is in two clinical trials focusing on safety, pharmacodynamics, suppression of spermatogenesis and hormones; the first of these two is estimated for completion in December 2024. There are also several non-hormonal methods with strong potential in preclinical stages of development.

**Conclusions:**

There exist several hurdles to novel male contraception. Therapeutic development takes decades of time, meticulous work, and financial investment, but with so many strong candidates it is our hope that there will soon be several safe, effective, and reversible contraceptive options available to male patients.

## Résumé

**Contexte:**

Depuis la sortie de la pilule contraceptive orale combinée en 1960, les femmes ont assumé le fardeau de la contraception et de la planification familiale. Plus de 60 ans plus tard, c’est toujours le cas, car les seules options contraceptives pratiques et efficaces disponibles pour les hommes sont les préservatifs et la vasectomie. Cependant, il existe maintenant une variété d’options contraceptives masculines hormonales et non hormonales prometteuses qui sont à l’étude. Le but de cette revue narrative est de fournir aux cliniciens et aux profanes des descriptions ciblées et à jour de nouvelles stratégies et cibles pour la contraception masculine. Nous incluons une discussion prudemment optimiste sur les avantages et les inconvénients potentiels, en soulignant plusieurs méthodes aux stades précliniques et cliniques du développement.

**Résultats:**

En juin 2023, deux méthodes contraceptives masculines hormonales faisaient l’objet d’essais cliniques de phase II pour leur innocuité et leur efficacité. Un essai international de phase IIb à grande échelle, portant sur l’efficacité de l’acétate de ségestérone transdermique (Nestorone) et du gel de testostérone, a recruté plus de 460 couples et devrait être achevé pour la fin de 2024. Une seconde méthode hormonale, l’undécanoate de diméthandrolone, fait l’objet de deux essais cliniques axés sur l’innocuité, la pharmacodynamique, la suppression de la spermatogenèse et des hormones; le premier de ces deux essais devrait être achevé en décembre 2024. Il existe également plusieurs méthodes non hormonales à fort potentiel aux stades précliniques de développement.

**Conclusions:**

Il existe plusieurs obstacles à la nouvelle contraception masculine. Le développement thérapeutique nécessite des décennies de temps, un travail méticuleux et un investissement financier ; mais avec autant de candidats solides, nous espérons qu’il y aura bientôt plusieurs options contraceptives sûres, efficaces et réversibles, disponibles pour les hommes.

## Introduction

In the wake of the *Dobbs v. Jackson Women’s Health Organization 2022* decision, the resultant “trigger laws” in 13 U.S. states, and the lingering retraction of reproductive rights in many more [1, 2], the need for novel contraceptive options has gained urgency across the United States. Unfortunately, due to a complex combination of medical challenges and societal beliefs [3–6], the burden of contraception has fallen almost entirely on women, and the only practical effective options available to males are condoms and vasectomy. Even with ‘perfect use’, the failure rate of condoms is still over 10% [7], and vasectomy is largely irreversible. Further, many of the contraceptive options currently available have high discontinuation rates [8], contributing to high rates of unintended pregnancy in the United States [9, 10]. With that in mind, there is a growing demand for safe, effective, and reversible male contraception that would allow men to share the burden of family planning [11, 12].

**Fig. 1:**
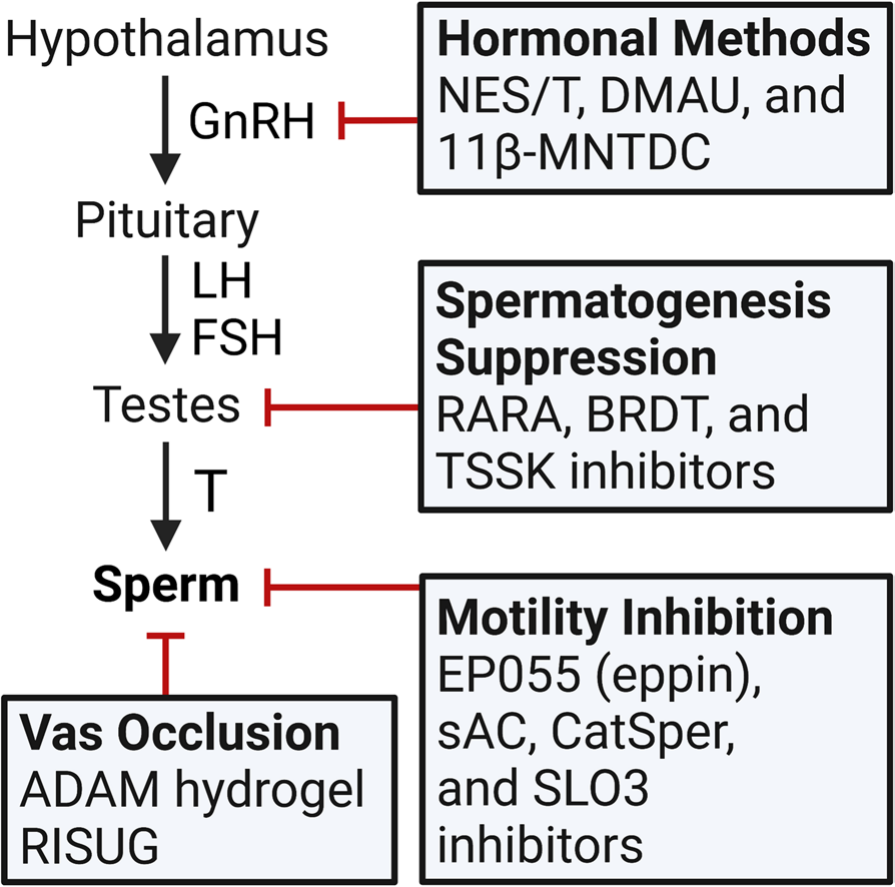
Overview of the hypothalamic-pituitary-testicular (HPT) axis and targets of male contraception. The HPT axis consists of the hypothalamus, pituitary gland, and testes. The hypothalamus releases gonadotropin-releasing hormone (GnRH) in a pulsatile fashion which signals for release of luteinizing hormone (LH) and follicle-stimulating hormone (FSH) from the anterior pituitary. LH and FSH drive testosterone (T) production and spermatogenesis in the testes. T and the hormonal contraceptives exert negative feedback on the hypothalamus to inhibit GnRH, LH, and FSH release, therefore suppressing spermatogenesis. Non-hormonal methods focus on distinct targets to inhibit spermatogenesis, sperm motility, or transit through the vas deferens. Pointed arrows indicate activation; red broad-tipped arrows indicate inhibition. NES/T, Nestorone/testosterone; DMAU, dimethandrolone undecanoate; 11β-MNTDC, 11β-methyl-19-nortestosterone dodecylcarbonate; RARA, retinoic acid receptor alpha; BRDT, bromodomain testis-specific protein; TSSK, testis-specific serine/threonine kinase; sAC, soluble adenylyl cyclase; CatSper, cation channel of sperm; SLO3, slowpoke homolog 3; RISUG, reversible inhibition of sperm under guidance. Figure created by EJL using BioRender.com

Male fertility is dependent on production of an adequate number of viable, motile sperm capable of moving through the female reproductive tract and fertilizing oocytes. Fertile males generally have seminal sperm concentrations greater than 15 million sperm/mL [13], and adequate sperm suppression for contraception requires sperm levels ≤ 1 million/mL [14]. The process of sperm production is termed spermatogenesis and is controlled by the hypothalamic-pituitary-testicular (HPT) axis (Fig. 1) [15]. Briefly, the hypothalamus produces gonadotropin-releasing hormone (GnRH) in a pulsatile fashion, which stimulates the anterior pituitary to secrete the gonadotrophic hormones luteinizing hormone (LH) and follicle-stimulating hormone (FSH). LH stimulates androgen production by testicular Leydig cells, and FSH, along with high levels of intratesticular T, enables spermatogenesis within the seminiferous tubules [16]. T exerts negative feedback on GnRH release and therefore suppresses LH and FSH secretion; the same effect is seen with exogenous androgens. Similarly, natural and synthetic progesterone, the latter termed progestins, exert negative feedback on the HPT axis to suppress LH and FSH release [16]. These concepts underlie the mechanisms of hormonal contraceptives discussed in this review, which generally target spermatogenesis, sperm motility, or transport through the vas deferens (Fig. 1).

There are several promising male contraceptive options in development, and they can be broadly categorized as either hormonal or non-hormonal. The purpose of this review is to provide an overview of the most promising male contraceptive methods under study, including how they work, their current state in research and development, and potential side effects or barriers to marketability. We will also briefly discuss some methods in preclinical stages of development to demonstrate that men may soon have access to a *variety* of safe, effective, and reversible contraceptive options.

## Materials and methods

For this narrative review, authors searched the online databases MEDLINE (via PubMed.gov), Cochrane Reviews, CENTRAL (via CochraneLibrary.com), ClinicalTrials.gov, and the World Health Organization’s International Clinical Trials Registry Platform for publications and ongoing clinical trials through 20 June 2023. Search terms included “contraception”, “male contraception”, “hormonal contraception”, “spermatogenesis inhibition”, “vas deferens occlusion”, and terms related to methods discussed below. Authors considered all identified ongoing studies related to male contraception, but we excluded from discussion those evaluating 7α-Methyl-19-nortestosterone (MENT) [17–19] or T combined with GnRH antagonists [20, 21], estradiol [22], or progestins (medroxyprogesterone acetate [23, 24] or norethisterone enanthate [25–28]) as these treatments ultimately failed to progress in clinical trials. Several past trials evaluating T injection alone [29–35] were also excluded as ongoing trials use T as a supplemental rather than primary compound. Authors EJL, GFLQ, and KLP completed literature search and assessed methodology of ongoing trials with particular focus on sample size (*n*), primary and secondary outcomes, and inclusion and exclusion criteria; none of the studies were excluded due to grossly unsound methodology.

### Hormonal methods

Three hormonal methods show great promise in male contraception: segesterone acetate (Nestorone; NES), dimethandrolone undecanoate (DMAU), and 11β-methyl-19-nortestosterone dodecylcarbonate (11β-MNTDC). NES and DMAU are currently in phase II clinical trials, and 11β-MNTDC has completed one phase II trial. Each method will be discussed separately below, and the clinical trials investigating these three compounds are summarized in Table 1.

**Table 1:** Summary of clinical trials investigating NES/T, DMAU, and 11β-MNTDC

	Study	Summary and outcomes
**NES/T**	Blithe and Myer 2023 [36, 37]	Ongoing phase II trial of 76–80 weeks daily topical NES/T for contraceptive efficacy; secondary outcomes include suppression of spermatogenesis, reversibility (recovery of spermatogenesis post-trial), hormone levels, mood symptoms, sexual side effects, and prostate function. *N* = 462 healthy couples. Primary endpoint completion estimated for September 2023 with full study completion by December 2024.
	Anawalt et al. 2019 [38]	Double-blinded phase I RCT of 28 days daily application of NES/T 8.3 mg/62.5 mg gel demonstrated gonadotropin suppression in 84% of participants without serious adverse events. *N* = 44 healthy males.
	Ilani et al. 2012 [39]	Double-blinded RCT of 6 months daily application of 10 g T with 0, 8, or 12 mg NES gel demonstrated sperm suppression in 23, 89, and 88% of men, respectively. T levels did not significantly change in any group, and adverse events were mild and minimal. *N* = 99 healthy males.
	Mahabadi et al. 2009 [40]	RCT of 20 days daily application of NES, T, or combination (NES + T). 92% of participants who achieved gonadotropin suppression had suppressed spermatogenesis after 3–4 weeks. Three subjects withdrew, but no serious adverse events were reported. *N* = 119 healthy males.
**DMAU**	Wang and Page 2023 [41]	Ongoing placebo-controlled, double-blinded phase I RCT of single IM (80-800 mg) or SC (50-200 mg) injection of DMAU assessing safety, tolerability, mood symptoms, sexual function, and overall health parameters. Secondary outcomes include suppression of gonadotropins, T, E2, SHBG, and spermatogenesis. *N* = 84 healthy males. Completion estimated for December 2024.
	Wang and Page 2020 [42]	Placebo-controlled, double-blinded phase II RCT of 12 weeks daily oral DMAU alone or with LNG after meal containing 25-30 g fat to assess suppression of spermatogenesis. Secondary outcomes include gonadotropin suppression, sperm counts, hormone changes, mood symptoms, sexual function, and overall health parameters. *N* = 100 health males. Publication of results is pending.
	Thirumalai et al. 2019 [43]	Placebo-controlled, double-blinded phase I RCT of 28 days daily oral DMAU from 100-400 mg after meal containing 25-30 g fat assessing safety, tolerability, and adverse events. Secondary outcomes included pharmacokinetics, pharmacodynamics, hormone changes, and sperm counts. DMAU suppressed T at even the lowest dose and caused dose-dependent suppression of LH and FSH. No serious adverse events observed. *N* = 82 healthy males.
	Ayoub et al. 2017 [44]	Placebo-controlled, double-blinded phase I RCT of single oral dose of 100-400 mg oral DMAU formulated in castor oil, self-emulsifying drug delivery system (SEDDS), or powder capsule fasted or following high-fat (50% calories as fat) meal assessing pharmacokinetics and pharmacodynamics, gonadotropin suppression, and hormone changes. High-fat meals increased absorption with all formulations, and DMAU exhibited dose-dependent suppression of gonadotropins, T, and E2 without serious adverse events. *N* = 44 healthy males.
	Surampudi et al. 2014 [45]	Placebo-controlled, double-blinded phase I RCT of single oral dose of 25-800 mg DMAU in powder capsule fasted or following high-fat (50% calories as fat) meal assessing pharmacokinetics, safety, and dietary fat’s effect on absorption. High-fat meals increased absorption, and DMAU exhibited dose-dependent suppression of gonadotropins, T, and E2 without serious adverse events. *N* = 19 healthy males.
**11β-MNTDC**	Yuen et al. 2020 [46, 47]	Placebo-controlled, double-blinded phase II RCT of single oral dose of 200 or 400 mg 11β-MNTDC assessing pharmacokinetics and pharmacodynamics. Secondary outcomes included gonadotropin suppression, hormone changes, mood, and sexual function. 11β-MNTDC exhibited dose-dependent suppression of gonadotropins and T with only mild side effects. *N* = 42 healthy males.
	Wu et al. 2019 [48]	Placebo-controlled, double-blinded phase I RCT of single oral dose of 100-800 mg 11β-MNTDC fasting or following high-fat (50% calories as fat) meal assessing receptor affinity. Secondary outcomes included safety, tolerability, pharmacokinetics, gonadotropin suppression, and hormone changes. 11β-MNTDC’s active metabolite demonstrated balanced affinity for androgen and progesterone receptors. 11β-MNTDC was well-tolerated and suppressed T without serious adverse events. *N* = 12 healthy males.

Trial summaries are listed by method in reverse chronological order

*NES/T* Nestorone/testosterone; *RCT* Randomized controlled trial; *DMAU* Dimethandrolone undecanoate; *LNG* Levonorgestrel; *IM* Intramuscular; *SC* Subcutaneous; *E2* Estradiol; *SHBG* Sex hormone binding globulin; *11β-MNTDC* 11β-methyl-19-nortestosterone dodecylcarbonate

#### Segesterone acetate + testosterone (NES/T)

Segesterone acetate, most often identified by its trade name Nestorone (NES), is a potent progestin with virtually no affinity for androgen receptors (AR) or estrogen receptors (ER) and minimal glucocorticoid activity [49–51]. NES shows low bioavailability when taken orally but is readily absorbed by transdermal application [52]; it has been available with ethinyl estradiol in the ANNOVERA vaginal ring (Mayne Pharma, Raleigh, NC) since 2018 and is a well-tolerated female contraceptive with > 97% efficacy [53–55]. NES is now compounded with T in a transdermal gel (NES/T) in a phase II clinical trial evaluating efficacy [36, 37]. T is added to improve suppression of spermatogenesis and minimize potential symptoms of androgen deficiency [56].

Phase I trials of NES/T daily gel (approximately 8.3 mg/62.5 mg) have demonstrated gonadotropin suppression adequate to suppress spermatogenesis in nearly 90% of participants [38, 39], suggesting that NES/T will be an effective form of male birth control [57]. Importantly, in these same studies there were no severe side effects with treatment. The main adverse effects were similar to the combined estrogen-progestin contraceptive pills used by women [58] and included minor mood symptoms, acne, and likely transient gastrointestinal symptoms [38–40]. From the most recent Phase I trial and a survey on attitudes towards NES/T, the majority of participants (79% and 56%, respectively) were satisfied or very satisfied with the treatments, and 50–51% reported that they would use NES/T daily gel as a sole form of contraception [38, 59].

A phase IIb trial investigating NES/T efficacy is currently underway at 17 medical centers across 8 U.S. states and 7 other countries [36, 37]; it is sponsored by the Population Council and the Eunice Kennedy Shriver National Institute of Child Health and Human Development (NICHD). Participants are self-administering the transdermal gel with one of two T doses (both compounded as NES/T; 8 mg/62 mg or 8 mg/74 mg), and participants showing low serum T with symptoms of hypogonadism will be offered additional T [37]. The trial is broken down into phases. There is an initial screening phase, after which participants begin daily NES/T. Within 20 weeks of beginning treatment, participants must show sperm suppression to levels ≤ 1 million sperm/mL before entering the 52-week efficacy phase. The recovery phase is intended to assess sperm production after ceasing NES/T application and continue symptom surveillance of both males and their female partners [37]. Enrollment was completed in November 2022 with 462 couples having started treatment. Completion of the primary endpoint, contraceptive efficacy, is estimated for late 2024, with full study results likely available in early 2025 [36, 37].

#### Dimethandrolone undecanoate (DMAU)

DMAU is a testosterone-derived pro-drug, metabolized to active form dimethandrolone (DMA), with high affinity for AR and, to a lesser degree, progesterone receptors (PR) [60]. DMAU and DMA are not aromatized and therefore lack estrogenic effects [61], but DMAU is highly lipophilic and experiences first-pass metabolism by the liver [62], requiring study of a variety of formulations to determine the optimal delivery method. In preclinical animal studies including non-human primates, DMAU was shown to effectively, reversibly suppress gonadotropins and spermatogenesis while maintaining physiologic androgenic effects without serious side effects [63–65]; importantly, there were no signs of liver toxicity, a well-characterized side effect of many exogenous androgens [66]. DMAU has been studied in several clinical trials for safety, pharmacodynamics, and gonadotropin suppression to evaluate its potential in male contraception, and phase I and phase II clinical trials are currently underway.

Early human trials of DMAU evaluated safety and absorption with doses up to 800 mg. In 2014, the first clinical trial orally dosed DMAU in a powder formulation from 25 to 800 mg, fasting or following high-fat meal (50% calories as fat). With a high-fat meal, authors found considerable, dose-escalating absorption of DMAU and suppression of gonadotropins (12 h later) from 200 mg upwards [45]. In a follow-up 2017 study, authors evaluated daily DMAU absorption at doses up to 400 mg daily and effects on estrogen and T levels [44]. Similarly, they found improved absorption with high-fat meals and suppression of estrogen and T in the absence of any serious side effects [44].

In a placebo-controlled, double-blinded, randomized phase I trial, Thirumalai et al. 2019 [43] investigated safety, tolerability, and adverse events associated with oral DMAU over 28 days of treatment, as well as pharmacokinetics, pharmacodynamics, hormonal changes, and sperm counts. The study found suppression of T at even the lowest dose of DMAU and dose-dependent suppression of LH and FSH, theoretically sufficient to suppress spermatogenesis with treatment for 10 weeks [57]. No serious side effects were observed; several participants reported decreased libido or erectile dysfunction, particularly at the highest tested dose, but participants did not report this affecting their sexual or erectile satisfaction [43]. Of note, DMAU was taken after a meal containing 25–30 g of fat, reflecting a typical Western diet but approximately half the fat content of the Ayoub et al. 2017 study [44]. In a secondary analysis of this trial’s samples and data, Thirumalai et al. 2021 found dose-dependent suppression of T and estrogen as well as an increase in a marker for bone formation over 28 days [67]. In another secondary analysis comparing metabolic effects of DMAU and 11β-MNTDC (discussed below), Yuen et al. 2021 found that DMAU caused a mean weight gain of 1.2 or 2.0 kg with 200 or 400 mg daily dosing, respectively, and mild lipid changes, but there were no serious adverse effects or signs of overt insulin resistance [46]. Collectively, these analyses indicate that orally dosed DMAU is well-tolerated and shows promise as a male contraceptive.

Today, there are two ongoing trials with DMAU, run by Drs. Christina Wang, MD, out of the University of California Los Angeles and Stephanie Page, MD, PhD, out of the University of Washington [41, 42]. Per ClinicalTrials.gov, both are reportedly still recruiting. The first is a phase I trial comparing a single injection of intramuscular (80-800 mg) vs. subcutaneous (50-200 mg) DMAU and is primarily assessing safety, pharmacodynamics, and hormonal suppression in healthy males [41]. Completion is estimated for December 2024. The second is a phase II trial primarily investigating the ability of orally dosed DMAU with or without a low dose of levonorgestrel (a progestin) to suppress spermatogenesis after 12 weeks treatment; secondary outcomes include hormonal suppression, serious adverse events, systemic symptoms, and tolerability [42]. Ideally, these ongoing studies will shed further light on the optimal route and dose of DMAU administration to guide efficacy trials.

#### 11β-methyl-19-nortestosterone dodecylcarbonate (11β-MNTDC)

11β-MNTDC is a testosterone derivative active at both AR and PR; it does not undergo aromatization and therefore lacks estrogenic effects [48, 61, 65]. Like DMAU, 11β-MNTDC is a pro-drug and is converted to 11β-methyl-19-nortestosterone (11β-MNT), which is structurally similar to DMA [68]. However, 11β-MNT’s affinity for AR and PR is more balanced than that of DMA (which favors AR), so side effect profiles may vary [48]. In preclinical animal studies, 11β-MNTDC was shown to effectively suppress serum gonadotropins [65] and exert even less liver toxicity than other androgens, including DMAU [63].

Several clinical trials have investigated 11β-MNTDC. The first major human trial was directed by Drs. Wang and Page and published in 2019 [48]. Twelve healthy adult males were given a single oral dose of 100-800 mg 11β-MNTDC with a high-fat meal or fasting, then assessed for pharmacokinetics, adverse effects, serum gonadotropins, and T levels. Like DMAU, 11β-MNTDC absorption was improved with high-fat meal, treatment was overall well-tolerated, and T was suppressed in a dose-dependent manner from 200 mg upwards [48]. Gonadotropin levels were not significantly reduced with a single dose, but this was addressed in a follow-up study published in 2020 [47]. This randomized, placebo-controlled phase II trial was again directed by Drs. Wang and Page, and participants received a daily oral dose of 200 or 400 mg 11β-MNTDC for 28 consecutive days. 11β-MNTDC was taken after a meal containing 25–30 g of fat [47], a more typical fat content per meal than in the previous trial [48]. Ultimately, 11β-MNTDC was well-tolerated; participants reported no serious adverse events, no one discontinued the trial due to side effects, and all reported side effects were mild or moderate. The most common sides effects were headache, acne, and decreased libido in 16% of participants [47]. Mood symptoms were reported, but they were comparable to those seen with currently available female estrogen-containing contraceptives [69–71]. 11β-MNTDC caused dose-dependent suppression of LH and FSH, and more participants in the 400 mg group had suppression to LH and FSH levels < 1.0 IU/L, the threshold at which spermatogenesis will be suppressed in nearly 90% of participants [57].

Efficacy trials are still needed for 11β-MNTDC, but between the two clinical trials and a secondary analysis comparing metabolic effects of DMAU and 11β-MNTDC (DMAU discussed above), 11β-MNTDC demonstrated acceptable safety profiles. Levels of T, estradiol, and sex hormone binding globulin (SHBG) were all suppressed, but these changes did not correlate with side effects or changes in serum chemistries [46–48]. 11β-MNTDC slightly increased participant weight and serum low-density lipoprotein (LDL) cholesterol levels, but there were no serious adverse events or signs of overt insulin resistance [46]. Results-to-date warrant clinical trials evaluating efficacy and safety using a larger number of participants.

### Non-hormonal methods

Several non-hormonal methods show promise in the field of male contraception, and two are either near human study or recently began human trials. In theory, these methods lack hormonal side effects, such as acne or mood symptoms, as well as the societal stigmas and false beliefs associated with hormonal contraception in the United States [6, 72]. The non-hormonal methods showing the most potential or closest to market, particularly those that inhibit spermatogenesis, motility, or vas deferens passage, will be discussed in greatest depth.

#### Spermatogenesis

All-trans retinoic acid (RA), also known as tretinoin, is derived from vitamin A and plays global roles in cell growth and development. RA plays essential roles in spermatogenesis and acts through binding the retinoic acid receptor alpha (RARA) located in the testes [73, 74]. The first human trial targeting RARA was conducted over 60 years ago with the non-selective RA biosynthesis inhibitor WIN 18,446 [75]. Sixty men were treated for one year, and spermatogenesis was suppressed in all participants throughout. However, off-target effects including inhibition of aldehyde dehydrogenase 2 in the liver unfortunately lead to a severe disulfiram-like reaction, effectively making the drug unmarketable [75]. Since then, the pharmaceutical company Bristol-Myers Squibb (BMS) designed and, with other labs, demonstrated effective, reversible suppression of spermatogenesis in mice with the pan-antagonist BMS-189,453 [76–78]. Theoretically, reversible alpha-selective agents would effectively and safely suppress sperm production without the systemic side effects of pan-antagonists. In other words, this would be an ideal method of contraception. Early attempts, most notably BMS-189,532 and BMS-189,614, lacked the efficacy of the pan-antagonist (WIN 18,446) by oral, intravenous, or intraperitoneal routes [79], but RARA remains a strong potential target for male contraception.

Bromodomains are amino acid segments in proteins that facilitate specific protein-protein interactions and a wide variety of cellular functions [80, 81]. One of these bromodomains, bromodomain testis-specific protein (BRDT), is required for spermatogenesis, and males with *BRDT* gene mutations are infertile with abnormal sperm morphology and impaired motility [82, 83]. Like RARA inhibition, specific inhibition of BRDT would theoretically suppress sperm production without the systemic effects of pan-inhibitors or hormonal methods. Indeed, inhibition of BRDT has been shown to effectively suppress spermatogenesis in male rodents using the small molecule JQ1 [84]. In this study, JQ1 was safe, reversible, and lacked obvious transgenerational effects, but authors noted potential off-target binding that could be reduced or prevented through design of more specific molecular inhibitors [84]. Progress has been made in the search for more specific BRDT inhibitors [85–88], but the compounds have yet to be tested in vivo and are therefore far from human trials.

Males express distinct testis-specific serine/threonine kinases (TSSK) that play spermatogenic roles in spermatids [89]. Mice with TSSK1 and TSSK2, TSSK3, or TSSK 6 deletions and human males with TSSK2 mutations are infertile, suggesting potential non-hormonal targets for contraception [90–93]. Of these, research into TSSK2 has shown the most progress. Since generation of enzymatically active, isolated TSSK2 [94], several inhibitors have demonstrated potent in vitro inhibition of TSSK2 [95]. To our knowledge, these inhibitors have yet to undergo in vivo study.

#### Motility

In order to reach and fertilize oocytes, sperm must travel through the female reproductive tract. This quality is termed *motility*, and immotile sperm are a major contributor to male-factor infertility [96]. Theoretically, by targeting enzymes or receptors that play essential roles in motility and are present only in sperm, one may reversibly immobilize sperm without systemic side effects. Eppin is an enzyme made in the testes that binds to the surface of sperm to play essential roles in motility [97]. Both immunization against eppin and molecular inhibition using the inhibitor EP055 has been shown to significantly, transiently reduce sperm motility [98, 99]. Although these studies were both done with small sample sizes and much work is needed before eppin inhibition may see clinical trials, no severe side effects were noted in these animal studies, suggesting that eppin may hold promise as a non-hormonal target [100].

In a similar vein as eppin, soluble adenylyl cyclase (sAC) is an intracellular signaling molecule needed for sperm capacitation, motility, and acrosome formation [101–103]. Several compounds have been tested in preclinical in vitro studies and shown to effectively inhibit sAC in mouse and human sperm [101, 104]. Indeed, sAC inhibition stands as a strong candidate for male contraception, and two recent studies have been conducted by Drs. Lonny Levin, PhD, and Jochen Buck, MD, PhD, out of Weill Cornell Medicine.

The first study by the Levin-Buck lab intricately compared capacitation and motility of sperm from sAC null mice and from healthy, wild type mice [105]. In vitro, they demonstrated that sAC plays essential roles in capacitation. In vivo, sAC null mice mated similarly to wild type mice, but their sperm were unable to migrate through the female reproductive tract. Essentially, these sperm were immotile [105]. Improving on the inhibitors mentioned above [102], a recent, well-designed study by the Levin-Buck lab investigated the new compound TDI-11,861; they demonstrated that a single oral or intraperitoneal dose of TDI-11,861 acutely inhibits sAC in mice, impairing capacitation and motility [103]. Importantly, the mice in this study had no changes in behavior, no obvious toxicity, and no pregnancies when treated within 2.5 h of mating [103]. With completion of this proof-of-concept study, authors anticipate additional safety and transgenerational studies to follow.

Calcium plays several signaling roles in sperm, including modulation of motility through activating sAC [96]. Extracellular calcium enters sperm flagella, the organelle that propels sperm, primarily through the cell type-specific cation channel of sperm (CatSper) [106]. Studies nearly 15 years ago demonstrated that immunologic inhibition of CatSper significantly suppresses sperm motility [107]; since, several compounds (RU1968 and HC-056456) have demonstrated effective inhibition of CatSper in vitro [108, 109] and preliminarily in vivo [110]. Several new compounds have been identified, synthesized, and tested on human sperm in vitro with excellent efficacy and safety profiles, at least on a cellular level [111]. Additional in vivo animal studies are anticipated.

Slowpoke homolog 3 (SLO3) is the main potassium channel in sperm and has functions directly related to calcium signaling and the CatSper channel [112, 113]. Like CatSper, SLO3 is specific to sperm and has functions essential for male fertility, making it an ideal target for male contraception [114–116]. A highly specific inhibitor of SLO3, VU0546110, has been identified and shown in vitro to inhibit sperm motility and acrosome reactions [117]. Better yet, at least one compound (termed “7 a” by Carlson et al. 2022) has been identified that blocks both SLO3 and CatSper, indicating potential for synergistic inhibition of sperm motility [111].

#### Vas deferens occlusion

The final target we wish readers to know about is physical obstruction of the vas deferens, termed ‘vas occlusion’, via gel injection to physically disrupt sperm during passage through the vas deferens. The benefits of this approach include fast installation (i.e. a quick injection at an outpatient visit) and relatively fast onset of action. A major barrier has been reversibility, but once overcome this approach may hold strong potential in male contraception. Several distinct polymers have been studied, including two styrene compounds termed “reversible inhibition of sperm under guidance” (RISUG) in India [118–120] and Valsalgel in the United States [121–123], and silicone and polyurethane compounds in the People’s Republic of China [124, 125]. The most recent trial of RISUG showed high contraceptive efficacy and a favorable safety profile [120], but human trials demonstrating reversibility of RISUG are needed. Despite these setbacks, one newer compound is being investigated in an ongoing clinical trial [126]. This new compound is a proprietary hydrogel, named ADAM by its founding company, Contraline Inc. of Charlottesville, Virginia. The trial started enrolling in late 2022 with a planned 25 total male participants through June 2025; ADAM injections will be done at the Epworth Freemasons Hospital in Melbourne, Australia. The primary outcome is adverse events, and secondary outcomes include percentage of participants achieving azoospermia and any serious adverse events [126].

### Limitations of the study

This review is subject to several limitations. The clinical trials discussed above are ongoing, and results have yet to be peer-reviewed and published. This does not yet allow for data-driven conclusions. Although this narrative review focuses on the most recent and ongoing studies of male contraception, authors recognize that it is not comprehensive. As mentioned above in Materials and Methods, several compounds were excluded because they failed to progress to human trials, failed after reaching human trials, or are in early preclinical stages. For these, we advise readers to explore several well-written reviews by Thirumalai and Amory [127], Long et al. [128], or the University of California San Diego urology department [129] that include many of these discontinued approaches.

## Conclusions

It is long overdue that male partners share the burden of family planning, and it is the authors’ hope that this will soon be a possibility. Ultimately, we feel that two of the methods discussed above—NES/T and DMAU—show the greatest potential for male contraception in the next decade. However, as clinical trials range from early planning stages to data collection stages, it may be several years before we see the efficacy and safety data needed to apply for FDA approval. In particular, the ongoing phase IIb NES/T trial results will not be published before 2025, and this is the method farthest along ‘the pipeline.’

Despite their many theoretical advantages to hormonal contraception, the non-hormonal targets are further from practical application. Authors recognize that there are many obstacles to reaching human studies, let alone late-stage clinical trials. Clinical trials require years of time, meticulous study, and financial support, and many compounds that perform well in pre-clinical animal studies fall short in human trials. The tools needed to efficiently design and study these non-hormonal targets are relatively young. However, they are already being employed to design and test strong drug candidates. As a society we now possess not only the scientific knowledge, technology, and clinical infrastructure needed to overcome these challenges, but also the social drive. With so many strong candidates, it is our hope that there will soon be several safe, effective, and reversible contraceptive options available to male patients.

## Data Availability

Not applicable.

## Abbreviations

11β-MNT: 11β-methyl-19-nortestosterone
11β-MNTDC: 11β-methyl-19-nortestosterone dodecylcarbonate
AR: Androgen receptor
BMS: Bristol-Myers Squibb
BRDT: Bromodomain testis-specific protein
CatSper: Cation channel of sperm
DMA: Dimethandrolone
DMAU: Dimethandrolone undecanoate
E2: Estradiol
FSH: Follicle-stimulating hormone
GnRH: Gonadotropin-releasing hormone
HPT: Hypothalamic-pituitary-testicular
IM: Intramuscular
LDL: Low-density lipoprotein
LH: Luteinizing hormone
LNG: Levonorgestrel
MENT: 7α-Methyl-19-nortestosterone
NES: Nestorone
NES/T: Nestorone/testosterone transdermal gel
NICHD: Eunice Kennedy Shriver National Institute of Child Health and Human Development
PR: Progesterone receptor
RA: All-trans retinoic acid
RARA: Retinoic acid receptor alpha
RCT: Randomized controlled trial
sAC: Soluble adenylyl cyclase
SC: Subcutaneous
SHBG: Sex hormone binding globulin
SLO3: Slowpoke homolog 3
T: Testosterone
TSSK: Testis-specific serine/threonine kinase
